# A green three-ratio manipulating spectrophotometric approaches for the determination of a binary mixture of pantoprazole and domperidone

**DOI:** 10.1186/s13065-025-01414-4

**Published:** 2025-03-03

**Authors:** Hamed H. M. Abuseada, Osama I. Abdel Sattar, Ahmed W. Madkour, Ahmed S. Taha

**Affiliations:** Pharmaceutical Analytical Chemistry Department, Faculty of Pharmacy, Al-Azhar University, Cairo, 11751 Egypt

**Keywords:** Spectrophotometry, Pantoprazole, Domperidone, Ratio difference, Ratio derivative, Mean centering, Green analytical chemistry

## Abstract

**Background:**

Pantoprazole (PAN) is a proton pump inhibitor used to treat GERD and hyperacidity by suppressing gastric acid secretion, effectively relieving symptoms such as heartburn, acid regurgitation, and indigestion. Domperidone (DOM) is a prokinetic agent that enhances gastrointestinal motility, helping to alleviate nausea, vomiting, and bloating caused by motility disorders. Their combination (Pantosec-D) provides rapid and comprehensive relief from both acid-related and motility-related symptoms, significantly improving patient comfort and quality of life.

**Objective:**

This study aims to develop and validate three eco-friendly spectrophotometric techniques—ratio difference (RD), first derivative (1DD), and mean centering (MC) of ratio spectra—for the simultaneous determination of PAN and DOM in pharmaceutical formulations.

**Method:**

The proposed methods resolve spectral overlap through ratio spectra manipulation. In the RD method, DOM is quantified by measuring the amplitude difference at 209 nm and 233 nm, while PAN is determined at 254 nm and 223 nm. The ^1^DD method detects DOM at 215 nm and PAN at 249 nm, whereas the MC method quantifies PAN at 254 nm and DOM at 209 nm.

**Results:**

The suggested methods were validated according to ICH regulations. Pharmaceutical formulations comprising PAN and DOM were effectively analyzed using the linear correlations obtained for both drugs over concentration ranges of 0.5–52 µg/mL and 1–18 µg/mL, respectively.

**Conclusion:**

Compared with reported spectrophotometric techniques, ratio methods are especially beneficial for routine pharmaceutical analysis due to their ease of use, capacity for handling overlapping spectra, and robustness to experimental variations. Compared with reported chromatographic methods, these techniques provide easy-to-use, reasonably priced, less solvent, and dependable substitutes for the standard quality control of these medications in pharmaceutical dosage forms.

**Supplementary Information:**

The online version contains supplementary material available at 10.1186/s13065-025-01414-4.

## Highlights

Three eco-friendly spectrophotometric techniques utilizing ratio spectra manipulation were developed to simultaneously determine pantoprazole and domperidone in their combined pharmaceutical tablet formulation.

**Supplementary Information:**

The online version contains supplementary material available at 10.1186/s13065-025-01414-4.

## Introduction

Pantoprazole (PAN) and domperidone (DOM) are commonly combined in pharmaceutical formulations to address gastrointestinal issues requiring both acid suppression and motility regulation [1–3]. PAN a proton pump inhibitor (PPI), treats conditions like GERD, peptic ulcers, and Zollinger-Ellison syndrome by irreversibly inhibiting the H + /K + -ATPase enzymes in stomach parietal cells. DOM a dopamine D2 receptor antagonist, enhances gastrointestinal motility and provides antiemetic effects by blocking dopamine receptors in the chemoreceptor trigger zone.

Pantosec-D tablets containing both pantoprazole and domperidone provide comprehensive treatment for motility and acid-related disorders.

Various analytical techniques, including HPLC, electrochemical methods, and spectrophotometry, have been widely reported for quantifying pantoprazole (PAN) in pharmaceutical formulations and plasma [4–8]. Similarly, domperidone (DOM) has been assessed using diverse methods such as spectrophotometry, electrochemical techniques, and HPLC [9–11].

Given the therapeutic importance of this drug combination, developing accurate and reliable methods for simultaneous quantifying PAN and DOM in pharmaceutical formulations is essential. Conventional techniques such as capillary electrophoresis, voltammetry, HPLC, and high-performance thin-layer chromatography (HPTLC) have been extensively utilized [12–17]. However, UV spectrophotometry remains the preferred method due to its simplicity, cost-effectiveness, and widespread availability in quality control laboratories.

**Fig. 1:**
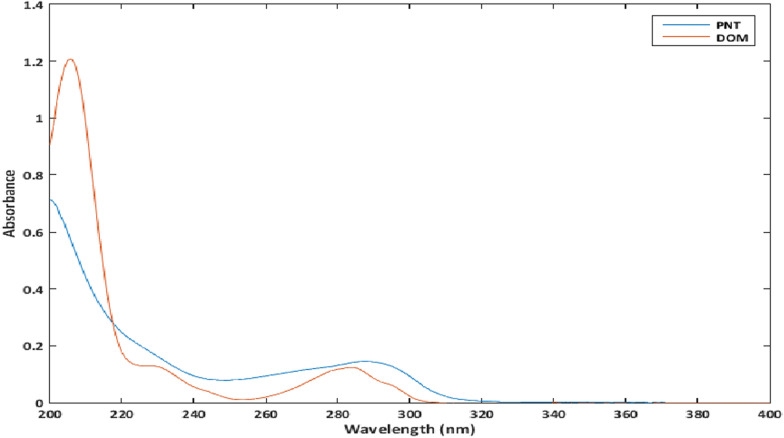
The absorption spectra were PNT (4 µg/mL) and DOM (4 µg/mL)

A significant challenge in the spectrophotometric analysis of multicomponent mixtures like PAN and DOM is the overlapping of their UV absorption spectra, which complicates their simultaneous determination (Fig. 1). Reported spectrophotometric methods [15] such as Q-analysis and simultaneous equations are limited by specific conditions, such as the need for iso-absorptive points, distinct absorption maxima, and minimal spectral overlap. These methods are also prone to errors and labor-intensive, making them less suitable for complex or multi-component formulations.

To overcome these limitations, advanced mathematical techniques have been employed to resolve spectral overlaps and enable precise quantification. Among these, the ratio difference (RD), first derivative (1DD), and mean centering (MC) methods have shown significant promise. These ratio-based approaches offer distinct advantages, including simpler calculations, better handling of overlapping spectra, reduced dependence on precise wavelength selection, suitability for complex mixtures, robustness to experimental variations, and minimized errors from instrument or sample variability. These features make ratio methods more practical and reliable for pharmaceutical analysis.

- *The RD Method*: This method evaluates the amplitude difference in the ratio spectra at specific wavelengths, effectively separating overlapping signals and allowing the independent quantification of each component in the mixture [18–23]^.^
- *The *^*1*^*DD Method*: By transforming the ratio spectra into their first derivatives, this method enhances the resolution of overlapping spectra, amplifying small differences and facilitating accurate discrimination and quantification of individual components [24–28].
- *The MC Method*: This approach centers the mean of the ratio spectra to zero, enhancing selectivity and accuracy. It has proven effective for the simultaneous evaluation of drugs in complex formulations, particularly where conventional methods fall short [29, 30].

This study aims to establish and validate these three spectrophotometric techniques—RD, 1DD, and MC—to quantify PAN and DOM in combined pharmaceutical formulations simultaneously. These approaches address the challenges posed by overlapping spectra and offer reliable alternatives to chromatographic methods.

The research optimized various parameters, including wavelength selection, solvent systems, and mathematical adjustments to the spectra, to ensure accuracy and precision. The efficacy and efficiency of these methods were demonstrated by comparing their results with a previously published spectrophotometric approach. Additionally, the study incorporated principles of green analytical chemistry, focusing on using fewer toxic solvents, minimizing chemical waste, and promoting eco-friendly practices in pharmaceutical analysis [31–34]. The Analytical Greenness (AGREE), ComplexMOGAPI, and NEMI tools were employed to evaluate the environmental friendliness of the developed techniques, The proposed methods achieved high results in greenness [35–41].

These optimized techniques ensure high accuracy and reliability while offering significant practical advantages for the simultaneous determination of DOM and PAN in combined pharmaceutical formulations. They provide a cost-effective, eco-friendly, and sustainable alternative for routine quality control in the pharmaceutical industry. With their ease of implementation, minimal solvent consumption, and operational efficiency, these methods are particularly well-suited for large-scale analysis of DOM and PAN formulations. By enabling precise quantification while minimizing environmental impact, they support regulatory compliance and promote sustainability in pharmaceutical testing.

## Experimental

### Materials

Cairo, Egypt-based MUP Medical Union Pharmaceuticals provides pure PAN (99.70%). Utilizing the reported method, the standard of purity was confirmed.

GlaxoSmithKline Pharmaceutical Industries, located in Cairo, Egypt, is the supplier of pure DOM (99.67%). The standard of purity was confirmed using the reported method.

### Pharmaceutical-preparation

*Pantosec-D* tab. was advertised as containing 10 mg of DOM and 40 mg of PAN. Manufactured under (batch no AFB23E23) by Cipla Ltd., Mumbai, India. And was purchased from the local market in India.

### Solvents

We utilized Analytical-grade ethanol from Sigma-Aldrich, Germany, and double-distilled water. Analytical-grade materials and reagents were used in the analysis techniques.

### Apparatus

Shimadzu UV–Visible 1800 spectrophotometer (Shimadzu Corp., Japan) was utilized for all measurements. Absorption spectra of samples have been scanned using quartz cuvettes 10 mm, and UV-Probe personal spectroscopy software version 2.21.

### Standard solutions

A pair of 100 mL volumetric flasks were used to prepare separate standard solutions of PAN and DOM (100 µg/mL). 10 mg of each medication standard powder was dissolved in 30 mL of ethanol. The solutions were thoroughly shaken, and ethanol was added to achieve the final volume.

### Procedures

- Linearity and calibration curves

Serial dilutions of PAN and DOM were prepared by transferring aliquots from their standard solutions (100 µg/mL) into separate 10-mL volumetric flasks, which were then filled with ethanol. This resulted in concentrations ranging from 2 to 48 µg/mL for PAN and 1–16 µg/mL for DOM. The absorption spectra of the various solutions (ranging from 200 to 350 nm) were measured using ethanol as the blank. To create ratio spectra, the absorption spectra of each drug were divided by the spectra of the other drug using the appropriate divisor spectrum. A 3 µg/mL solution of DOM was used to generate the PAN ratio spectra, while a 10 µg/mL solution of PAN was used for the DOM ratio spectra.

1. *RD Technique*: To generate calibration graphs and formulate regression equations, the difference in the amplitude values of the ratio spectra at 254 and 233 nm for PAN and at 209 and 233 nm for DOM were plotted against the respective concentrations of each medication.
2. ^*1*^*DD Technique*: Using Δλ = 8 nm and a scaling factor of 40, the ratio spectra of both medications were converted to their first-order derivatives. The ^1^DD amplitude values for DOM and PAN were obtained at 215 nm and 249 nm, respectively. Calibration graphs were created by plotting the recorded data against the drug concentrations in µg/mL, and the relative regression equations were then derived.
3. *MC Technique*: To improve accuracy and selectivity, the mean-centered ratio spectra of each medication were calculated using MATLAB. The mean-centered amplitude values for DOM and PAN were determined at 209 nm and 254 nm, respectively. Regression equations were then derived by plotting these values against the relevant concentrations of each drug to build the calibration curves.
4. Application to Lab. prepared mixture

Five samples were prepared using 10 mL volumetric flasks containing aliquots of the DOM and PAN standard solutions at different concentrations (1:4), which were subsequently diluted with ethanol to volume. The procedure described for each technique's linearity and calibration curves was followed to analyze the samples and determine the concentration of both drugs.

iii. Pharmaceutical application

Ten Pantosec-D tablets, each containing 40 mg of PAN and 10 mg of DOM, were ground into a fine powder after being weighed. A 100 mL volumetric flask was filled with 40 mL of ethanol and an exact portion of the powdered material (equivalent to one tablet). After 20 min of vigorous shaking, the flask was filtered and filled with ethanol to the 100 mL mark. The solution was then diluted with ethanol to provide five distinct concentrations. The samples were analyzed according to the procedures outlined for each technique, using the linearity and calibration graphs, to determine the concentrations of each medication.

### Outcome and discussion

Due to a significant overlap in their typical UV absorption spectra, measuring PAN and DOM simultaneously is challenging. Three ratio spectra manipulation techniques—RD, 1DD, and MC—were employed to overcome the overlap issue and quantitatively identify PAN and DOM in their combination tablets. To obtain the ratio spectra of the drugs under study, each drug's normal spectrum was first divided by a fitting spectrum of the other drug, which served as the divisor. It is crucial to select a divisor that enhances both the signal-to-noise ratio and sensitivity, so various PAN and DOM spectra were considered for use as divisors. A 3 µg/mL DOM solution was the ideal divisor for generating PAN ratio spectra. Conversely, for the DOM ratio spectra, a 10 µg/mL solution of PAN was optimal.

**Fig. 2:**
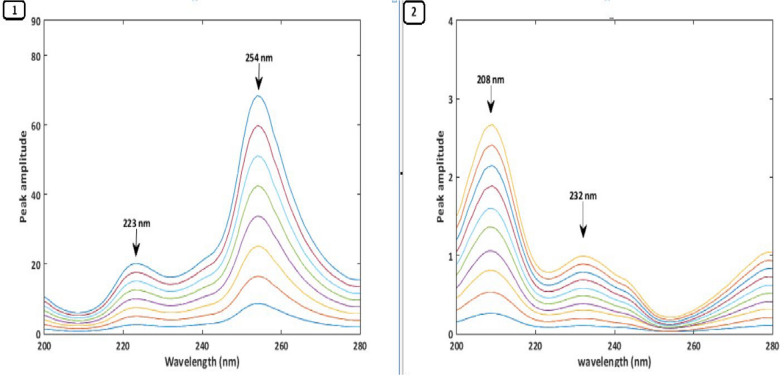
**1** Ratio spectra of PAN (6–48 µg/mL), **2** Ratio spectra of DOM (2–11 µg/mL)

**Fig. 3:**
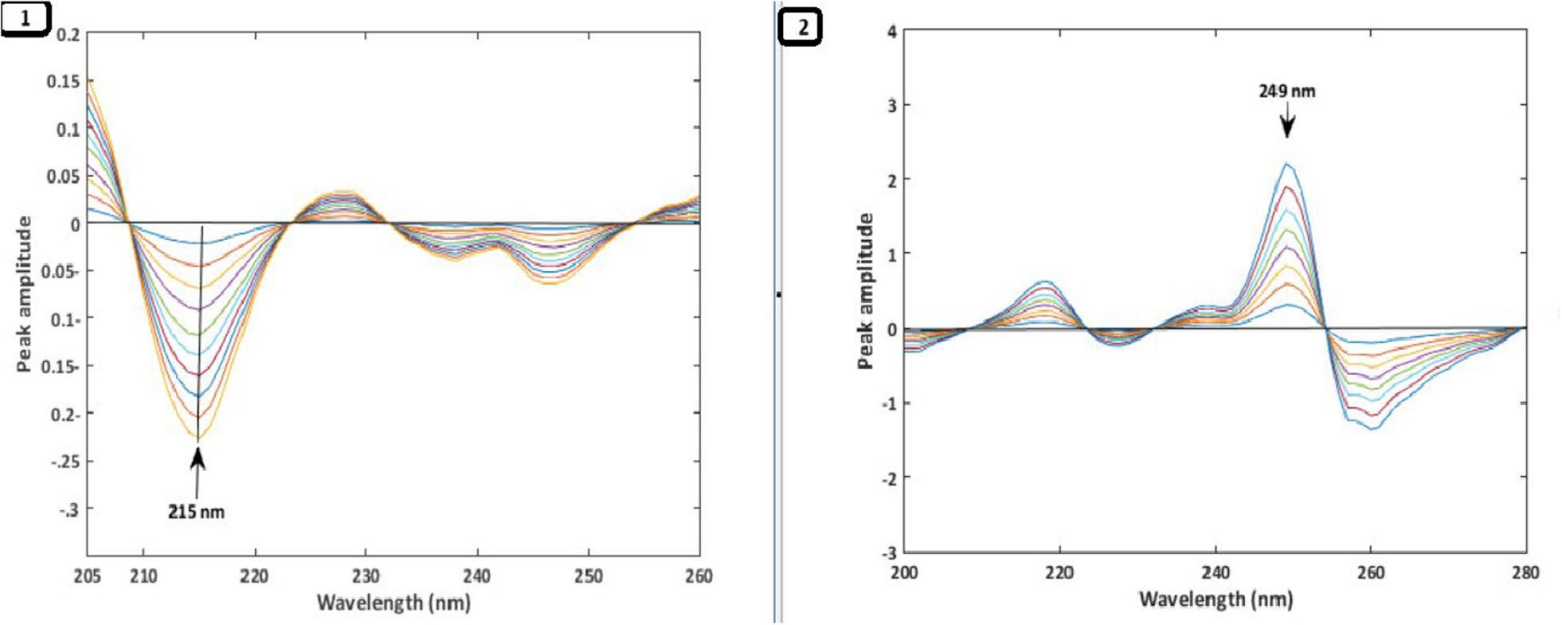
**1** ^1^DD spectra of DOM (2–11 µg/mL), **2** ^1^DD spectra of PAN (6–48 µg/mL)

**Fig. 4:**
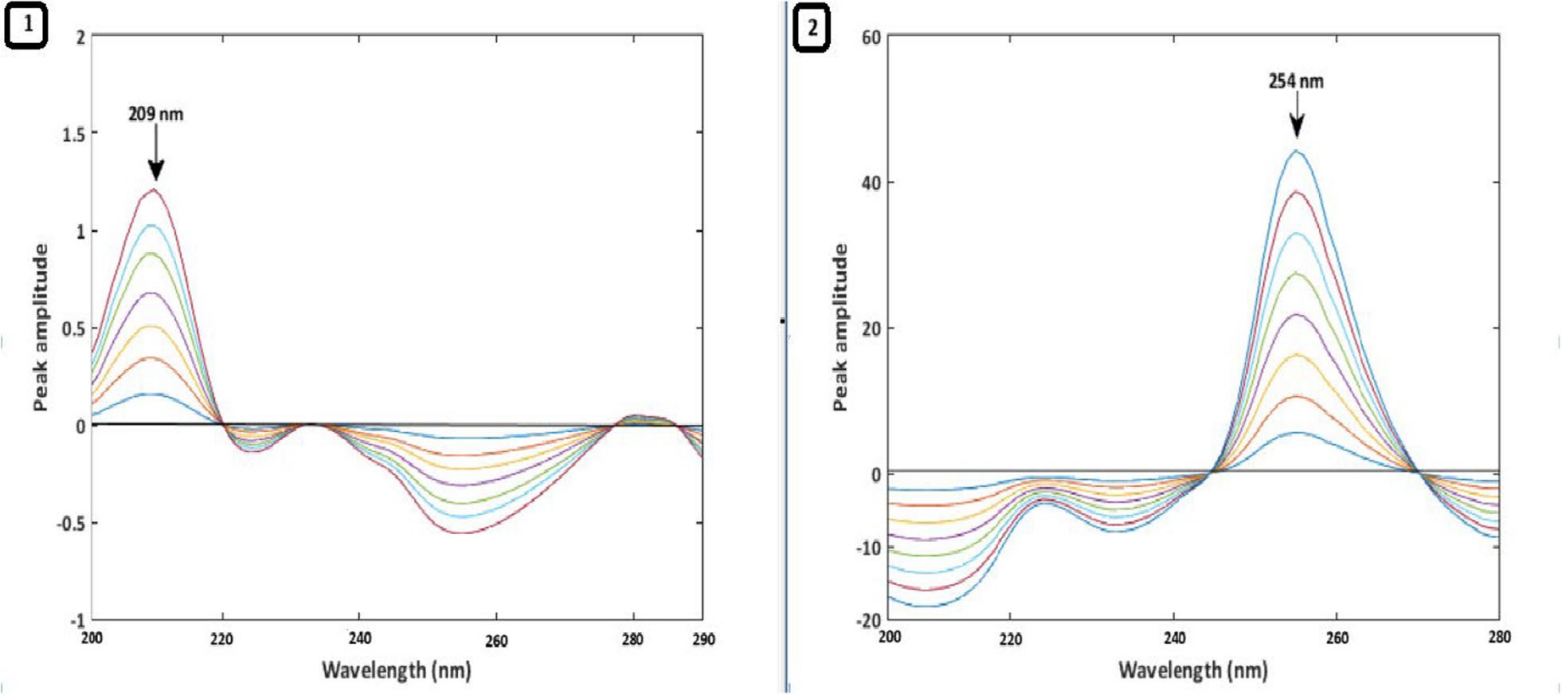
**1** MC spectra of DOM (2–14 µg/mL), **2** MC spectra of PAN (6–48 µg/mL)

A. *RD Method*: To apply the RD method, it is essential to compute the amplitude values at the selected wavelength pairs. Therefore, the linearity at each selected wavelength was investigated. It was found that the PAN ratio spectra showed good linearity at 254 nm and 233 nm, while the DOM ratio spectra showed good linearity at 209 nm and 233 nm. As a result, PAN could be determined independently in the combination without interference from DOM by calculating the difference in amplitude values between 254 and 233 nm. Similarly, DOM could be measured separately in the mixture by calculating the difference in amplitude values between 209 and 233 nm, without interference from PAN (Fig. 2). Calibration curves were generated by plotting the difference in amplitude values at predefined wavelengths for PAN and DOM against each drug concentration in µg/mL. The concentrations of each drug in the combination were then calculated using regression equations.
B. ^*1*^*DD Method*: The ratio spectra of each drug were converted into their first-order derivative using a scaling factor of 40 and Δλ = 8 nm. An evaluation of the linearity and selectivity of the ^1^DD spectra of the two compounds at different peak amplitudes showed that the wavelengths of 249 nm for PAN and 215 nm for DOM exhibited excellent linearity and selectivity. Thus, PAN could be measured in the mixture at 249 nm without interference from DOM, and DOM could be measured at 215 nm without interference from PAN (Fig. 3).
C. *MC Method*: In the mean-centering method, the ratio spectra of each drug were mean-centered using MATLAB to enhance the resolution and selectivity of the overlapping signals. Evaluation of the mean-centered spectra showed that PAN could be accurately quantified at 254 nm, while DOM could be accurately quantified at 209 nm. This indicates that PAN and DOM can be determined in the mixture at 254 nm and 209 nm, respectively, without interference (Fig. 4). The Analytical Greenness (AGREE), ComplexMOGAPI, and NEMI tools were applied to assess the environmental impact of the established techniques. The proposed methods exhibit high results for greenness Table 1.

**Table 1:** Comprehensive greenness evaluation of the proposed and reported methods

Proposed methods	Reported methods
NEMI tool	NEMI tool
	
AGREE tool	AGREE tool
	
ComplexMOGAPI tool	ComplexMOGAPI tool
	

### Validation

The ICH guidelines were followed to validate the procedures [42]. These procedures' linearity, range, accuracy, precision, limit of detection (LOD), limit of quantitation (LOQ), selectivity, and robustness were assessed. The findings showed adequate linearity in the PNT and DOM concentration ranges of 0.5–52 µg/mL and 1–18 µg/mL, respectively. Comprehensive regression analysis data are given in (Table 2). The slope of the calibration curve and the regression line's residual standard deviation were used to determine the LOD and LOQ values. The techniques demonstrated their sensitivity, yielding LOD values of 0.621 µg/mL for DOM and 0.209 µg/mL for PNT in the RD method, 0.625 µg/mL for DOM and 0.211 µg/mL for PNT in the 1DD method, and 0.624 µg/mL for DOM and 0.209 µg/mL for PNT in the MC method.

1. Accuracy and precision

**Table 2:** Regression and validation data for the determination of pantoprazole and domperidone by the proposed methods

Parameters	RD		^1^DD		MC	
	PNT	DOM	PNT	DOM	PNT	DOM
Wavelength (nm)	254–223	208–232	249	215	254	209
Linearity range (μg/mL)	0.5–52	1–18	0.5–52	1–18	0.5–52	1–18
LOD (μg/mL)	0.209	0.621	0.209	0.625	0.209	0.624
LOQ (μg/mL)	0.442	0.912	0.443	0.913	0.439	0.916
Slope (b)	1.0104	0.1706	0.9694	0.0116	1.4331	0.2694
Intercept (a)	− 0.2941	− 0.0032	0.2557	0.0007	− 0.4035	− 0.2742
Coefficient of determination (r^2^)	0.9998	0.9998	0.9999	0.9998	0.9999	0.9999
Accuracy (%R)^a^	100.39	100.17	99.72	99.96	100.23	99.35
Repeatability precision (RSD)^b^	1.176	1.141	0.976	0.959	1.509	1.178
Intermediate precision (RSD)^b^	1.001	0.919	0.965	0.847	0.79	1.133
Robustness^c^ (%RSD) Wavelength (± 1 nm)^c^	1.251	1.181	1.061	0.972	1.372	1.206

^a^Nine measurements were averaged (three times in three different concentrations)

^b^%RSD of nine measurements (three times in three different concentrations)

^c^%RSD of determination of three concentrations of each drug after slight changes in the wavelength (± 1 nm)

The mean percent recovery (%R) was computed using triplicate measurements at three concentration levels within the linearity range for both drugs: 3, 6, and 9 µg/mL for DOM and 5, 15, and 30 µg/mL for PNT. The results showed that the mean percent recovery values for both drugs were within the acceptable range of 98% to 102%, confirming the accuracy of the developed techniques.

Precision was evaluated by calculating the relative standard deviation (RSD). For repeatability, RSD values were less than 2% when three duplicate measurements were taken on the same day. Similarly, intermediate precision, assessed over three different days, also exhibited RSD values below 2%, meeting the acceptability criterion. The outcomes, displayed in Table 2, validate the precision and accuracy of the established methods.

2. Specificity and selectivity

The methods demonstrated excellent specificity and selectivity, as no interference was observed from tablet excipients or other additives during the assessment of PNT and DOM in laboratory-prepared solutions (Table 3) and Pantosec-D tablets (Table 4). Selectivity was confirmed using the standard addition procedure, which verified the accurate detection of PNT and DOM within the tablet matrix. The results complied with the criteria for specificity and selectivity, confirming that the methods could reliably distinguish and quantify PNT and DOM without interference.

3. Linearity and range

**Table 3:** Determination of pantoprazole and domperidone in synthetic laboratory mixtures by the proposed methods

Added (µg/mL)		RD		1DD		MC	
PNT	DOM	PNT	DOM	PNT	DOM	PNT	DOM
8	2	100.77	99.05	99.39	98.76	100.52	98.61
16	4	98.12	99.83	100.02	101.24	100.64	101.67
24	6	99.91	98.7	99.46	99.82	101.35	99.73
32	8	101.35	99.34	100.18	98.91	98.97	100.22
40	10	100.83	101.42	101.09	100.85	99.13	100.47
Mean ± %RSD		100.22 ± 1.423	100.13 ± 1.192	99.80 ± 0.699	100.26 ± 1.164	100.29 ± 0.947	100.45 ± 1.052

**Table 4:** Determination of pantoprazole and domperidone in Pantosec-D® tablets by the proposed and the reported methods

	Mean ± %RSD							
	PAN				DOM			
	RD	1DD	MC	Reported method	RD	1DD	MC	Reported method
Pantosec-D tab.^a^	100.92 ± 0.984	100.22 ± 0.614	99.35 ± 1.423	101.09 ± 0.823	100.63 ± 0.676	100.14 ± 1.568	99.22 ± 1.422	100.87 ± 1.1.069
Standard addition^b^	99.32 ± 0.845	100.53 ± 0.749	100.61 ± 0.918	–	99.20 ± 1.347	100.11 ± 0.785	100.22 ± 0.669	–
Student’s *t*-test^c^ (2.306)	0.300	0.297	2.294	–	0.425	0.855	2.066	–
*F*-value^c^ (6.388)	1.425	1.794	3.156	–	2.513	2.123	1.766	–

^a^Five measurements were averaged

^b^Three measurements were averaged

^c^The numbers in parenthesis are the “t” and “F” tabulated values at (P = 0.05)

The methods demonstrated excellent linearity across the specified concentration ranges: 0.5–52 µg/mL for PNT and 1–18 µg/mL for DOM. The Ring Bom optimum concentration ranges were identified as 2–44 µg/mL for PNT and 3–15 µg/mL for DOM, indicating the regions of highest accuracy and precision. Regression analysis revealed correlation coefficients (R^2^) greater than 0.999 for both drugs, which is within the accepted value for linearity. These results confirm that the methods are suitable for quantifying PNT and DOM over the specified ranges.

4. Sensitivity (LOD and LOQ)

Sensitivity was evaluated by calculating the limit of detection (LOD) and the limit of quantitation (LOQ). The LOD values were 0.621 µg/mL for DOM and 0.209 µg/mL for PNT in the RD method, 0.625 µg/mL for DOM and 0.211 µg/mL for PNT in the 1DD method, and 0.624 µg/mL for DOM and 0.209 µg/mL for PNT in the MC method. Similarly, the LOQ values were sufficiently low to ensure accurate quantification within the specified ranges. These values confirm the methods' high sensitivity, meeting the acceptance criteria for detecting and quantifying PNT and DOM.

5. Robustness

The methods' robustness was tested by introducing small, deliberate changes to experimental conditions, such as detection wavelength. These variations did not significantly affect the outcomes, demonstrating the methods' reliability. All results were consistent with the acceptable variation limits for robustness, ensuring that the techniques remain effective under varied conditions. Table 2 shows that the process was robust because the percentage RSD of the answers was less than 2%.

## Conclusion

In this work, we have developed three environmentally friendly UV spectrophotometric techniques for the quantification of domperidone (DOM) and pantoprazole (PNT) in combined pharmaceutical products (RD, ^1^DD, and MC). These techniques were specifically designed to address the issue of overlapping absorption spectra of DOM and PNT. The methods demonstrated high sensitivity, with LOD values of 0.621 µg/mL for DOM and 0.209 µg/mL for PNT using the RD method, 0.625 µg/mL for DOM and 0.211 µg/mL for PNT in the 1DD method, and 0.624 µg/mL for DOM and 0.209 µg/mL for PNT in the MC method.

Compared to previously reported spectrophotometric techniques [15], ratio methods offer distinct advantages, particularly in routine pharmaceutical analysis. They are easy to use, effectively handle overlapping spectra, and demonstrate robustness against experimental variations.

In terms of practical significance, this work introduces simple and eco-friendly spectrophotometric methods for the simultaneous determination of PAN and DOM in pharmaceutical formulations, without the need for complex instrumentation. These methods offer a cost-effective and accessible alternative to more advanced analytical techniques, making them particularly suitable for routine pharmaceutical analysis and quality control. Their simplicity, sensitivity, and selectivity ensure reliable quantification while minimizing environmental impact, thereby strengthening their role in sustainable pharmaceutical testing.

## Supplementary Information

Supplementary Material 1

## Data Availability

Data is available upon request.
