# Supplementary material for: A green three-ratio manipulating spectrophotometric approaches for the determination of a binary mixture of pantoprazole and domperidone
Source: BMC Chem. 2025 Mar 3;19(1):57. doi: 10.1186/s13065-025-01414-4 (PMC11874381; doi:10.1186/s13065-025-01414-4)
Supplement: Supplementary file 1 — Supplementary Material 1 [file 13065_2025_1414_MOESM1_ESM.docx]

Figure: S1 shows the structural formulas for DOM and PNT


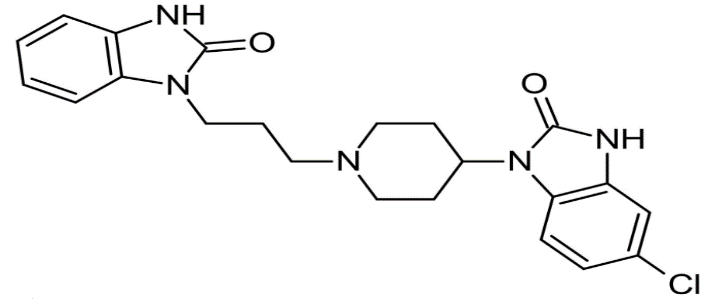
 PNT DOM


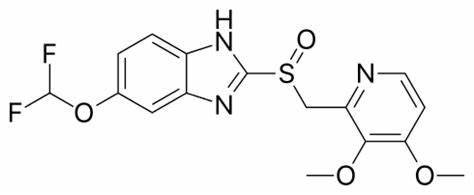


**Comprehensive greenness evaluation:**

Recent trends in analytical chemistry focus on incorporating sustainability concepts into method development. Evaluating the sustainability of analytical methods is crucial for understanding their environmental impacts.

Greenness assessment was conducted using the National Environmental Method Index (NEMI), Complementary Green Analytical Procedure Index (Complex GAPI), and Analytical Greenness Metric (AGREE) tools.

**Evaluation of the method's greenness profile:**

- **NEMI tool:**

NEMI tool provides a qualitative, simple, yet effective visual greenness assessment based on key parameters like toxicity, corrosiveness, and waste generation. NEMI leverages pictograms containing four key criteria, as shown in **Figure (S2)**, which are considered green if specific criteria are met. These criteria include: (1) chemicals employed in this approach not being classified as persistent, bio-accumulative, and toxic (PBT) as per the Toxic Release Inventory (TRI) and Agency Environmental Protection Agency's (EPA), (2) The chemicals employed in this procedure are not categorized as hazardous based on the criteria set forth by the (RCRA), specifically in terms of their classification under the U, P, F, D, or TRI lists of potentially hazardous waste, (3) The method's pH level is non-corrosive, falling within the range of 2 to 12, while (4) the amount of generated waste remains under 50 grams. In this work, NEMI pictograms were established, as shown in Table (5). The suggested method immediately stood out as a green method since four quadrants were colored green, satisfying four NEMI criteria. The pH of the methodology is 6.5, so it is non-corrosive as well as the amount of waste that is produced is fewer than 50 g. Overall, while NEMI provides an initial broad screen of a method's general greenness, recent studies have highlighted some reliability concerns for performing greenness assessments using this tool alone due to the simplified pass/fail approach employed by NEMI based on a limited set of criteria. Therefore, in the current work, we apply NEMI as an initial first screen but combine it with more robust quantitative greenness metrics.

Figure (S2): Typical NEMI pictograms.


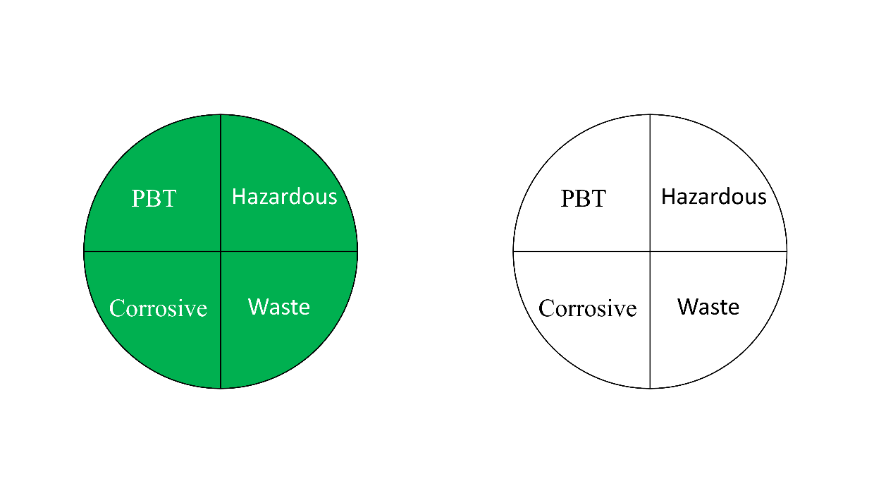


- **ComplexMOGAPI tool:**

ComplexMOGABI is a powerful tool for evaluating the sustainability of analytical methods, providing a detailed and balanced assessment. It builds on the original MOGABI framework by adding an advanced scoring system that looks at both the environmental and economic aspects of a method. This means it doesn’t just assess how green a method is but also considers how efficient and cost-effective it is. ComplexMOGABI evaluates every stage of the workflow—from method development and sample handling to reagent use, instrument operation, and waste management.

One of the standout features of ComplexMOGABI is its intuitive visual representation. Using a simple color-coded system (green for sustainable, yellow for moderate concern, and red for challenges), makes it easy to understand the sustainability of a method at a glance. Additionally, key metrics like energy use and cost-per-analysis are calculated, with lower values indicating better efficiency and savings.

In this work, ComplexMOGABI revealed that the developed method is exceptionally sustainable. Most of the indicators were in the green zone, and the energy consumption and operational costs were impressively low. Table (4).

- **AGREE tool:**

AGREE metric provides another valuable quantitative approach for greenness assessment based on the 12 principles of green analytical chemistry (GAC). A key advantage of AGREE is the ability to assign custom weighting factors to parameters based on their relevance to the specific analytical method. This enables customized assessments focused on the most pertinent greenness criteria. The final AGREE score from 0 to 1 summarizes the overall method greenness, while the visual clock pictogram identifies areas needing improvement and can be conveniently applied using freely available software. In this work, AGREE analysis for the suggested method was done. Based on the evaluation using AGREE, the suggested method exhibits outstanding greenness, achieving a high score (0.82), indicating its more efficient performance in terms of green principles. The graphs presented in **Table (4)** visually demonstrate the remarkable greenness of the suggested method, reinforcing its environmentally friendly characteristics and confirming its alignment with eco-friendly analytical practices. However, AGREE is limited to environmental criteria related to green chemistry principles. Other key sustainability dimensions like safety, performance, and cost-effectiveness are not addressed. Thus, coupling AGREE with complementary tools assessing other sustainability dimensions is recommended for a more well-rounded assessment. Overall, AGREE is a valuable tool but should be part of a combined approach to guide the advancement of greener, more sustainable analytical techniques.

Table S1. Green assessments comparison and results of the proposed methods using AGREE

| AGREE Metric | Description |
| --- | --- |
| 1. Sample Treatment | Sample treatment in (At- line analysis) |
| 2. Minimal Sample Size | Minimal sample size and minimal number of samples (1 mL) |
| 3. In Situ Measurements | In situ measurements (At-line) |
| 4. Integration of Processes | Integration of processes saves energy and reduces reagent use (one step used) |
| 5. Automated Methods | Semi-automated and miniaturized methods |
| 6. Derivatization | No derivatization needed |
| 7. Waste Management | Volume and management of analytical waste (1 mL) |
| 8. Multianalyte Methods | Multianalyte methods are preferred over single-analyte methods at a time. |
| 9. Energy Use | Energy use should be minimized (<0.1 kWh per sample UV-Vis spectrophotometer) |
| 10. Renewable Reagents | Reagents from renewable sources should be preferred (ethanol only solvent used) |
| 11. Toxic Reagents | Toxic reagents should be eliminated or replaced (no toxic reagent used) |
| 12. Operator Safety | Operator safety should be increased |
| A score of 0.82 indicates that the spectrophotometric methods were green | |
